# Effects of Graphene Nanoplatelet Size and Surface Area on the AC Electrical Conductivity and Dielectric Constant of Epoxy Nanocomposites

**DOI:** 10.3390/polym10050477

**Published:** 2018-04-27

**Authors:** Anil Raj Ravindran, Chuang Feng, Shu Huang, Yu Wang, Zhan Zhao, Jie Yang

**Affiliations:** 1 Sir Lawrence Wackett Aerospace Research Centre, School of Engineering, RMIT University, GPO Box 2476, Melbourne, VIC 3001, Australia; anil.ravindran@rmit.edu.au; 2 School of Engineering, RMIT University, Bundoora, VIC 3083, Australia; shu.huang@rmit.edu.au (S.H.); s3415279@student.rmit.edu.au (Y.W.); s3494699@student.rmit.edu.au (Z.Z.)

**Keywords:** graphene platelet, epoxy nanocomposites, electrical conductivity, dielectric constant

## Abstract

Epoxy nanocomposites reinforced with various grades of multilayer graphene nanoplatelets (GNPs) are manufactured and tested. The effects of size, surface area, and concentration of GNP, as well as alternating current (AC) frequency on the electrical and dielectric properties of epoxy nanocomposites are experimentally investigated. GNPs with larger size and surface area are always beneficial to increase the electrical conductivity of the composites. However, their effects on the dielectric constant are highly dependent on GNP concentration and AC frequency. At lower GNP concentration, the dielectric constant increases proportionally with the increase in GNP size, while decreasing as the AC frequency increases. At higher GNP concentration in epoxy, the dielectric constant first increases with the increase of the GNP size, but decreases thereafter. This trend is also observed for varying the processed GNP surface area on the dielectric constant. Moreover, the variations of the electrical conductivity and dielectric constant with the GNP concentration and AC frequency are then correlated with the measured interfiller spacing and GNP diameter.

## 1. Introduction

Two-dimensional graphene and its derivatives have attracted tremendous interest, due to their excellent mechanical and physical properties. These properties, including high Young’s modulus (~1 TPa), high thermal conductivity (5000 W m^−1^ K^−1^), high electrical conductivity (~6 × 10^5^ S m^−1^), make graphene and its derivatives promising material candidates for extensive applications [1,2,3,4,5]. Applications range from adding graphene into polymer matrices to develop high performance multifunctional composites to super capacitors [6]. Studies have demonstrated that a small amount of graphene added into polymers can improve the overall mechanical and physical properties without reducing the beneficial attributes of the polymers [7,8,9,10,11,12,13,14,15,16]. For example, Rafiee et al. [17] demonstrated that the Young’s modulus of epoxy nanocomposites can be increased by 31% by adding 0.1 wt % graphene nanoplatelet (GNP). Compared to pure polymer, an increase of up to 121% in thermal conductivity was observed by Kim et al. for GNP-reinforced polymer composites [18]. More work on evidencing the reinforcing effects of graphene on mechanical, thermal performance, and functionality of composite materials and structures can also be found in [12,13,14,15,16,19,20,21,22,23,24,25,26,27,28,29,30,31,32,33,34,35].

Recently, developing polymer composites with enhanced electrical conductivity and dielectric constant (relative permittivity) has stimulated a surge in academic and industrial communities, due to their great potential in applications such as high-storage capacitors, electromagnetic shielding, and artificial muscles in electrostriction systems [36,37]. The use of carbon nanotubes (CNTs) and/or GNPs as the conductive fillers in polymer composites have been extensively studied [36,38,39,40,41,42,43,44,45,46,47,48,49,50,51,52,53,54]. An electrical conductivity of 2.11 S/m was achieved by Zhang et al. [55] with an addition of only 3.0 vol % of graphene into polyethylene terephthalate (PET). Cui et al. [56] prepared graphene/PVDF composites and reported significant increase in electrical conductivity. A maximum dielectric constant of 2080 was achieved at 1000 Hz with 12.5 vol % graphene dispersed in PVDF. Yousefi et al. [57] developed highly aligned graphene/polymer nanocomposites with significantly improved electrical conductivity and excellent dielectric properties. For example, by adding 3 wt % of reduced graphene oxide (rGO), they obtained exceptionally high dielectric constant of over 14,000 at 1 kHz. Pan and co-workers [58] obtained highly flexible graphene/poly(vinylidene fluoride) (PVDF) composites with high dielectric constant, i.e., more than 340 at 100 Hz.

Thermosetting epoxies are widely used for applications ranging from structural adhesives to coatings for engineering structures. The use of epoxy polymers as a matrix has garnered significant attention in the fabrication of advanced polymer composites. The insulating nature of epoxy composites results in inferior resistance to damage and poor electrical discharge properties when subjected to electrical loads [59]. Examples of these events may include lightning strike damage on fiber-reinforced epoxy composites structures, inducing delamination damage, that further degrades the mechanical properties [60,61,62]. This poses a challenge where highly electrically conductive epoxies are desired. Although a lot of effort has been devoted to studying the electrical properties of graphene-reinforced composites, to the best of the authors’ knowledge, limited work has been found on the investigating the effect of the size and surface area of graphene towards the AC electrical conductivity and dielectric constant of the epoxy nanocomposites. Therefore, in the present study, epoxy nanocomposites containing multilayered GNPs at varying geometries are manufactured and tested, to investigate the dependency of AC electrical conductivity and dielectric constant of the nanocomposites on the applied electrical field. Firstly, the effect of filler content at a selected GNPs geometry is investigated towards the electrical properties described above. Secondly, the influence of GNP surface area (i.e., XGNP C-series) and diameter (i.e., XGNP H-series) on the electrical properties is parametrically investigated. Lastly, the enhancements of GNP towards the electrical properties are then correlated with the spacing between fillers along with the measured size of the GNPs in epoxy via scan electron microscopy. 

## 2. Materials and Experimental Details

### 2.1. GNP and Epoxy Materials 

Commercially available GNPs that were used in manufacturing the nanocomposites within this study were supplied by XG Sciences at different grades (i.e., C and H series). The C grade GNPs that were employed included XGNP^®^ C-300, C-500, and C-750 series containing an average particle diameter of less than 2 µm and supplied surface areas of 300 m^2^ g^−1^, 500 m^2^ g^−1^, and 750 m^2^ g^−1^, respectively. The H grade GNPs incorporated were XGNP^®^ H-5, H-15, and H-25, with an average supplied surface area of 60 to 80 m^2^ g^−1^ and average particle diameter of 5 µm, 15 µm, and 25 µm, respectively. Note that the surface area of the GNP is the value from the grouping of the GNP (i.e., C-series) specified by the supplier. The supplied GNPs have a thickness ranging between 1–20 nm. The GNPs were doped into liquid epoxy resin at different concentrations to manufacture the nanocomposites. The liquid epoxy resin used within this study was a two-part system containing a liquid blend of bisphenol A and bisphenol F (“105” from West System) and slow hardener (“206” from West System). The blend was of aliphatic amines and aliphatic amine adduct based on diethylenetriamine and triethylenetetramine [63]. A 2.5 mm thick mastic tape was used as dam to cure the epoxy nanocomposites over a glass bench.

### 2.2. Nanocomposite Fabrication

**Figure 1:**
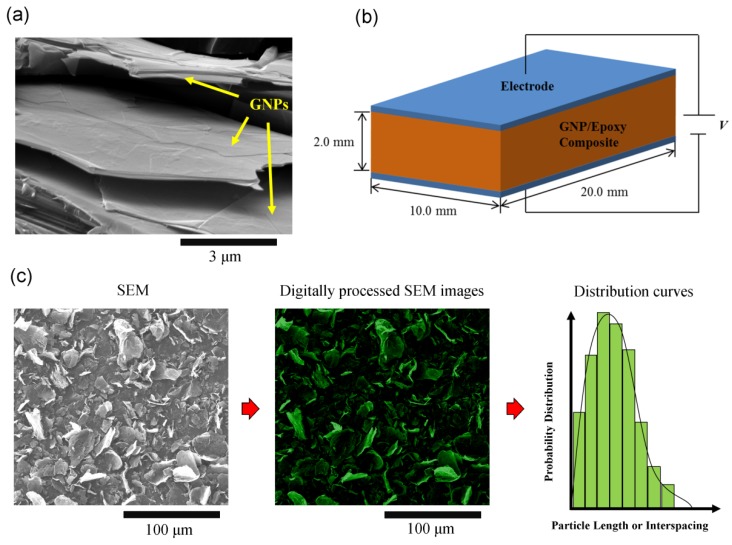
(**a**) Scanning electron microscope (SEM) image of graphene nanoplatelet (GNP) (H-15 Series, 5 wt % in epoxy). (**b**) Specimen configuration of GNP/epoxy composite used for electrical conductivity and relative permittivity measurements. (**c**) Process of attaining the GNP length and interfiller spacing from SEM images (H-15 Series, 5 wt % in epoxy) followed by digital image correlation (DIC).

The GNP fillers were first mixed with the liquid epoxy in high speed mixer for 30 min at 500 rpm to allow even homogenization of the nanofillers. Prior to adding hardener, a three-roll mill (Dermamill 100) was then employed to disperse the GNPs in liquid epoxy. The three-roll milling process was conducted by passing the mixture 10 times at 150 rpm with a roller-gap distance of 20 µm, which functions to calendar and exfoliate GNP aggregates, to ensure good dispersion of the fillers. Further details of the three-roll milling mixing process are adapted from [10,47,64,65]. In depicting the surface morphology of the GNPs after milling, Figure 1a presents a scanning electron microscope (SEM) image of the milled GNP along the fracture surface of the epoxy. Following the three-roll milling process, hardener was added to the epoxy resin mixture stoichiometrically (i.e., 5:1, ratio of epoxy resin to hardener) in consideration of the filler content. The mixture, along with the hardener, was mixed manually for 5 min, followed by degasification for 5 min to minimize the voids. The GNP/epoxy mixture was then poured into a mastic mold that was sized to manufacture epoxy nanocomposite samples with length, width, and thickness being 200 mm, 100 mm, and 2.5 mm, respectively. The samples were then cured for 48 h at room temperature (i.e., 25 °C) in accordance with resin supplier’s guidelines [63]. For the first study in investigating the effect of GNP content upon the dielectric constant and AC conductivity of the epoxy, GNP C-300 was used at concentrations of 0.0, 0.10, 0.50, 1.0, 1.5, 2.0, 5.0, 10.0, and 20.0 wt %, respectively. For assessing the influence of surface areas on the properties, GNP C-300, C-500, and C-750 were employed at weight fraction 5.0 wt % and 10.0 wt %, respectively. The addition of GNP H-5, H-15, and H-25 into epoxy was carried out at concentrations of 1.0, 5.0, and 10.0 wt % GNP, respectively, in measuring the AC electrical conductivity and dielectric constant.

### 2.3. Sample Fabrication and Test

Cured GNP/epoxy nanocomposites were cut and polished into 10 mm wide, 20 mm long, and 2 mm thick samples, as shown in Figure 1b. A diamond saw was used to cut along width and length directions, while the top and bottom surfaces of the samples were grinded and polished to the desired thickness, i.e., 2.0 ± 0.1 mm using a roll grinder with a 600 grit aluminum oxide grit paper. Samples were then blown with compressed air, and degreased with acetone to remove surface impurities. Before sticking aluminum electrodes, conductive silver paste was coated on both surfaces of the length and width plane. In connection with the electrodes, the AC conductivity and dielectric permittivity along the through-the-thickness direction of the nanocomposites were measured by impedance analyzer (HP4190A). For these properties, AC frequencies ranging between 10^3^ Hz to 10^7^ Hz and 5 Hz to 10^7^ Hz are selected, respectively. For each configuration, a minimum of three samples were tested to obtain averaged data. All measurements were conducted at room temperature in accordance with ASTM D3380-14 [66].

### 2.4. Scanning Electron Microscopy for Evaluation of Interfiller Spacing and Filler Diameter

The dispersion of GNP in cured GNP/epoxy nanocomposites was assessed by performing profilometry analysis via high resolution scanning electron microscopy (SEM). SEM revolves around the process of scanning the surface of a substrate with a focused beam of electrons, thereby interacting with the atoms in the surface of the samples to generate a variety of signals in producing an image based on the external morphology, chemical composition, structure, and orientation of materials (i.e., features). SEM samples of the GNP/epoxy composites were prepared by immersing bar-type samples in liquid nitrogen. Samples were then snapped off to allow a brittle fracture to investigate the surface morphology of the samples along the thickness and width plane. Samples were then surface-coated with a thin layer of gold prior to observation. SEM analysis was conducted under a FEI Nova NanoSEM operated at 10–15 kV and 5 mm working distance. In measuring the minimum distance between the neighboring GNPs (i.e., GNP interspacing or interfiller spacing) along the fracture surface of the epoxy composite, six high resolution SEM images were captured with an area of 50 µm by 50 µm. In performing the quantitative measurements, digital image correlation (DIC) and processing software, ImageJ, was used. For accurate processing, as presented in Figure 1c, a sharpening and change in contrast was performed to emphasize graphene fillers from the epoxy in the fracture surface of the SEM images, containing GNPs at various contents, supplied surface areas and supplied filler diameters. The fillers, highlighted in green, within the filtered images were selected. The distribution on the GNP interspacing and diameter was measured using the DIC and processing software. Transmission electron microscopy (TEM) was considered in assessing the dispersion state of the GNPs added into the epoxy. However, in consideration to ultramicrotomy process in preparing the samples for imaging, TEM may not be appropriate for imaging micron size features, particularly, for the epoxy nanocomposite containing GNPs at diameters ranging from 15 µm to 25 µm at various orientation distribution investigated within this study (i.e., H-15 and H-25 series). A significant amount of time would be required to accurately stitch various captured TEM micrographs and digitally analyze the geometric distribution in length and interfiller spacing. To ensure consistency, the use of the high-resolution SEM techniques described above was performed. This SEM technique of quantitatively assessing the dimensions, distribution, volume fraction, and interspacing of the fillers is adapted from [64,67,68].

## 3. Results and Discussion

### 3.1. Effect of GNP Content on AC Electrical Conductivity and Dielectric Constant

**Figure 2:**
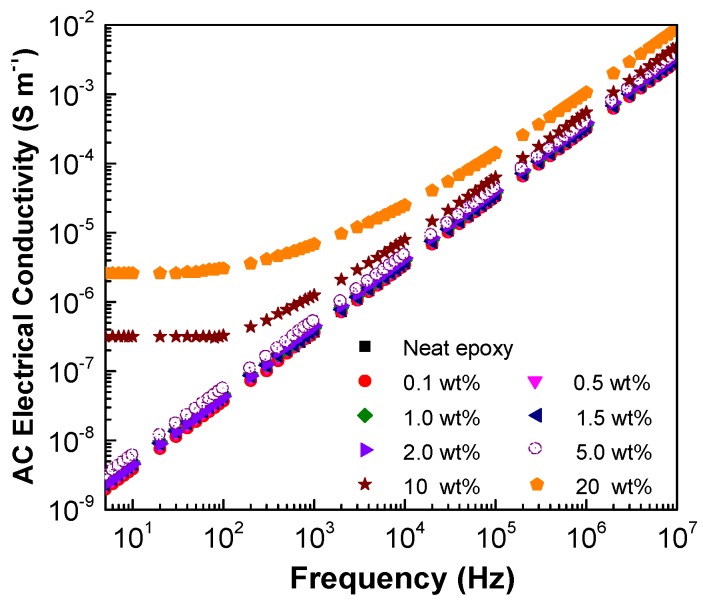
AC electrical conductivity of GNP/epoxy nanocomposites with varying content of GNP with a surface area of 300 m^2^ g^−1^.

**Figure 3:**
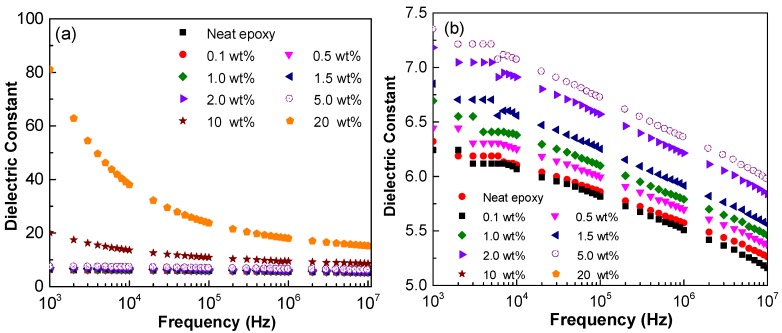
(**a**) Dielectric constant of GNP/epoxy nanocomposites with concentration GNP C-300; (**b**) Inset of Figure 3a.

Figure 2 and Figure 3 present the AC electrical conductivity and dielectric constant plots as logarithmic functions of frequency with increasing content of GNP C-300 in epoxy. As shown in Figure 2, the AC conductivity increases with the electric frequency. This can be attributed to the fact that as the frequency increases, more electrons are conducted across the interface between the GNP and the polymer matrix due to the frequency facilitated electron hopping. It is also found that for lower GNP fractions ranging between 0.1–5.0 wt % in epoxy, limited discrepancy can be found between the AC conductivity with increasing conductivity for frequencies above 5 Hz. However, for higher GNP concentrations at 10 wt % and 20 wt %, a plateau in the AC electrical conductivity is presented at lower frequencies ranging between 5–100 Hz. However, the AC conductivity curves converge together while frequency is further increasing. Such phenomena can be explained by the formation of percolation networks and attributes of the electrical conductivity of the interface between the GNP and the polymer matrix, which consists of static (i.e., DC conductivity) and AC frequency dependent parts [69],

(1) $$\sigma^{*}=\sigma +i\omega \varepsilon$$

where σ^*^ is the overall electrical conductivity, ***σ*** is the static part and *iωε* represents the frequency dependent part with *ω* being the AC frequency and *ε* being the dielectric permittivity. When the AC frequency is low, the frequency dependent part of electrical conductivity is not significant. The electrical conductivity of the interface is dominated by the static part, which is closely related to filler concentration and distance between neighboring GNPs. For lower GNP concentration, i.e., 0.1–5.0 wt %, large separation distance exists between neighboring GNPs, resulting in negligible increase in the static part of the electrical conductivity. As the frequency increases, the frequency facilitated electron hopping dominates the electrical conductivity over the static electrical conductivity. Therefore, the difference among the curves with various filler concentration becomes limited.

The variations of dielectric constant with frequency are shown in Figure 3. Although the dielectric constant decreases with the increase of the AC frequency, a similar trend is observed for the dielectric constant as that for the electrical conductivity. The dielectric constant for neat epoxy is 6.3 at 10^3^ Hz, as it is consistent to values (i.e., approximately 6) reported in a previous study using the same epoxy system [70]. The value for epoxy containing 5.0 wt % GNP C-300 is found to be 7.3, indicating limited reinforcing effects on the dielectric properties. When the GNP concentration increases to higher value, such as 10 wt % and 20 wt % in epoxy, a significant difference between the dielectric constant at lower AC frequency is observed. With increasing frequency, the dielectric constant with various GNP concentrations converges together. This is because as the concentration is low, with no percolated networks, i.e., 0.1–5.0 wt %, there is limited Maxwall–Wagner–Sillars (MWS) polarization effect [69], which is believed to be the mechanism of dielectric properties of the nanocomposites. Therefore, it results in negligible increase in the dielectric constant. For higher filler concentration, the electrons accumulated at the interface between the GNP and the polymer matrix reduces remarkably as the AC frequency increases, leading to the decrease in the dielectric constant.

**Figure 4:**
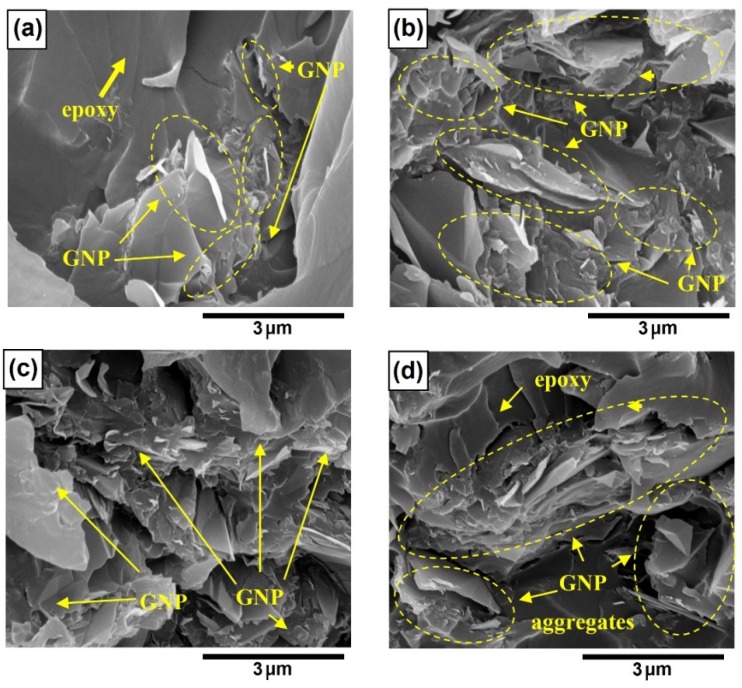
SEM of fracture surface of GNP (C-300 series) in epoxy at loadings of (**a**) *f*_GNP_ = 1.0 wt %; (**b**) *f*_GNP_ = 2.0 wt %; (**c**) *f*_GNP_ = 10 wt %; (**d**) *f*_GNP_ = 20.0 wt %.

**Figure 5:**
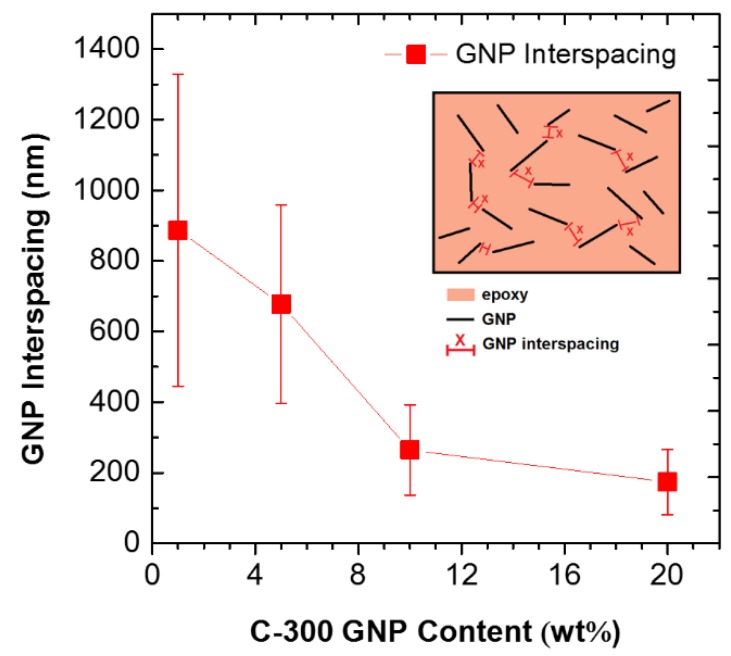
GNP interspacing after milling at varying GNP (C-300 series) content in epoxy nanocomposite.

The above observations on the electrical conductivity and the dielectric constant can be further evidenced by the SEM fractography, as shown in Figure 4. As indicated by the circles, some GNPs were observed on the fracture surface as shown in Figure 4a,b. The protruded GNPs were originated from debonding of GNP from the polymer matrix upon failure. The fractured surfaces become much coarser when GNP loading increases to 10 wt % and 20 wt % (see Figure 4c,d). Figure 5 presents the GNPs interspacing in epoxy with increase in GNP content in epoxy. This indicates stronger epoxy/GNP interaction at higher GNP concentration. In addition, owing to the graphene platelet-to-platelet interactions, the increase in conductivity and dielectric constant is attributed to the increased packing density and reduced spacing between the neighboring platelets. The stronger epoxy/GNP interaction, together with platelet-to-platelet interactions of graphene, may lead to the formation a greater density of the GNP percolation networks. Therefore, a significant increase of electrical conductivity was observed for GNP/epoxy nanocomposites with GNP loading at 10 wt % and 20 wt %. 

### 3.2. Effect of GNP Surface Area on AC Electrical Conductivity and Dielectric Constant

**Figure 6:**
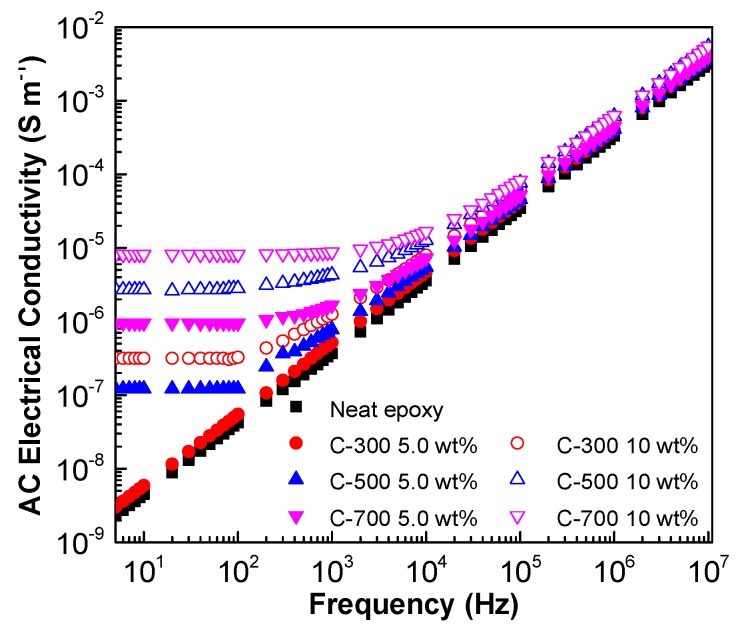
AC electrical conductivity of GNP/epoxy nanocomposites at varying supplied GNP surface area (Note: the surface area of the GNP is the value from the grouping of the GNP (i.e., C-series) specified by the supplier).

**Figure 7:**
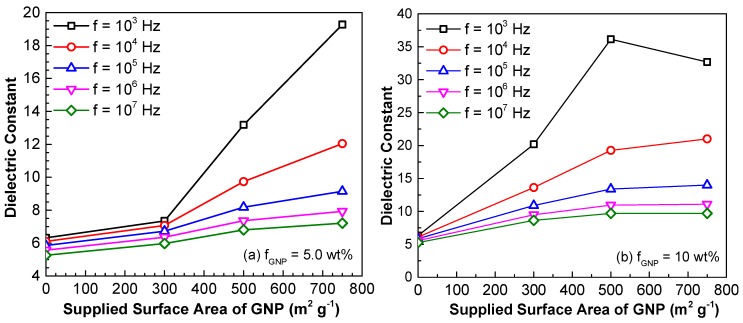
Dielectric constant of GNP/epoxy nanocomposites with different GNP weight fractions at (**a**) 5.0 wt % and (**b**) 10 wt % for varying supplied GNP surface area. (Note: the surface area of the GNP is the value from the grouping of the GNP (i.e., C-series) specified by the supplier).

Figure 6 and Figure 7 present the AC electrical conductivity and dielectric constant of the nanocomposites with different supplied GNP surface areas and concentrations. As shown in Figure 6, the electrical conductivity increases with the increase of the AC frequency and GNP concentration. Increasing the surface area of the GNPs results in an increase in the AC electrical conductivity, especially when the frequency is relatively low, i.e., 5–10^3^ Hz. This can be correlated the reduced GNP interfiller spacing, due to larger surface area as argued by Noh et al. [68]. Moreover, the increased surface area of the GNPs may allow for a greater degree of exfoliation of the stacked graphene sheets during the three-roll milling process, leading to a greater chance of forming percolated networks.

Figure 7 demonstrates the effect of supplied GNP surface area on the dielectric constant of the nanocomposites with 5 wt % and 10 wt % of GNPs, respectively. As observed, the dielectric constant decreases with the increase of the AC frequency for all cases. However, when the concentration is 5 wt % (see Figure 7a), the dielectric constant increases with the increase of the surface area and such an increase is more sensitive to the GNPs with larger surface area. At 10 wt % GNP in epoxy (see Figure 7b), the dielectric constant enhances with the increase of the surface area, as the AC frequency is greater than 10^4^ Hz. However, when the frequency is 10^3^ Hz, the dielectric constant decreases as the surface area is larger than 500 m^2^ g^−1^. This sudden decrease may indicate that at lower AC frequency, the nanocomposite is transitioning from an insulator to conductor when a considerable amount of GNPs with large surface areas are dispersed into polymers. 

**Figure 8:**
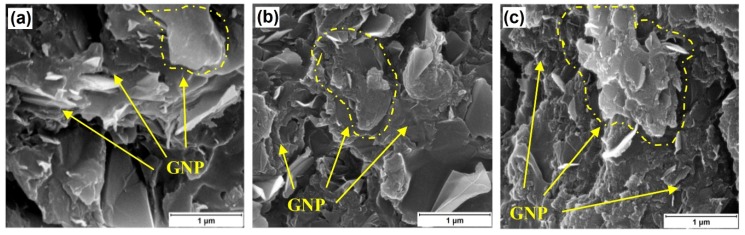
SEM of fracture surface of GNP/epoxy nanocomposite containing 10 wt % GNP at (**a**) C-300; (**b**) C-500; (**c**) C-750.

**Figure 9:**
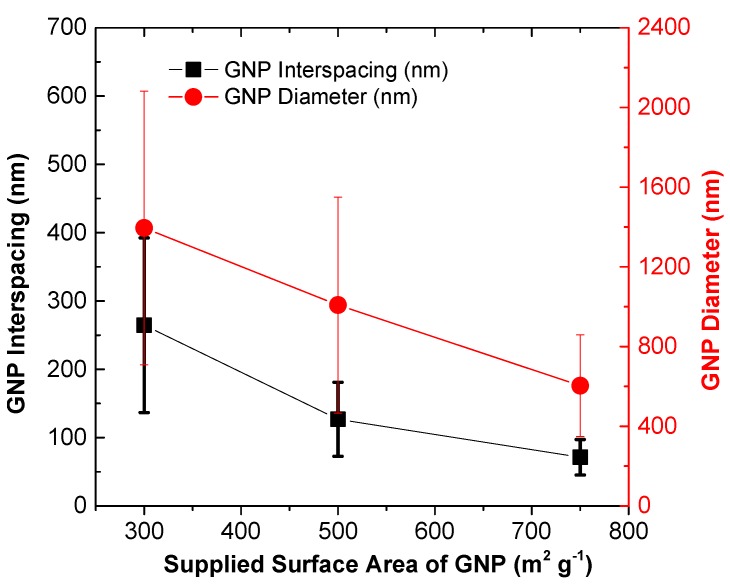
GNP interspacing and diameter after milling at varying supplied GNP surface area in epoxy nanocomposite containing 10 wt % GNP. (Note: the surface area of the GNP is the value from the grouping of the GNP (i.e., C-series) specified by the supplier).

Figure 8 demonstrates the SEM fractograph of the epoxy nanocomposites reinforced by GNPs at varying supplied surface areas. With the increase in the supplied GNP surface area, the GNP aggregates contain more features and greater degree of GNP exfoliation within the epoxy fracture surface. In measuring the interfiller spacing between the neighboring GNPs, Figure 9 shows the GNP interspacing and milled diameter of GNPs at 10 wt % in epoxy with increase in GNP surface area. With the increase in GNPs surface area increases, there is a reduction in the interfiller spacing and milled diameter. The reduction in diameter of GNP can be attributed to shearing of the GNP aggregates in epoxy resin during the three-roll milling process. This may indeed correlate to increased exfoliation of the GNPs aggregates at higher surface area, which contains more short stacks of graphene sheets and process induced geometric defects [28,69]. These defects along the thickness of the multilayer platelets would act as localized stress raiser, enabling the GNPs to be more susceptible to mechanically induce exfoliation during any shear-based mixing process [10,47,71]. Accordingly, this results in greater number of GNPs within the vicinity and increased density of the percolating networks. The reduced interfiller spacing with the increase in GNP surface area does correlate well with the increased AC electrical conductivity and dielectric constant, as observed. GNPs with smaller diameter can be well dispersed in polymer matrix with less curvature and agglomerations, which is advantageous to enhance the electrical conductivity of the nanocomposites to some extent [68,71]. 

### 3.3. Effect of GNP Size on AC Electrical Conductivity and Dielectric Constant

**Figure 10:**
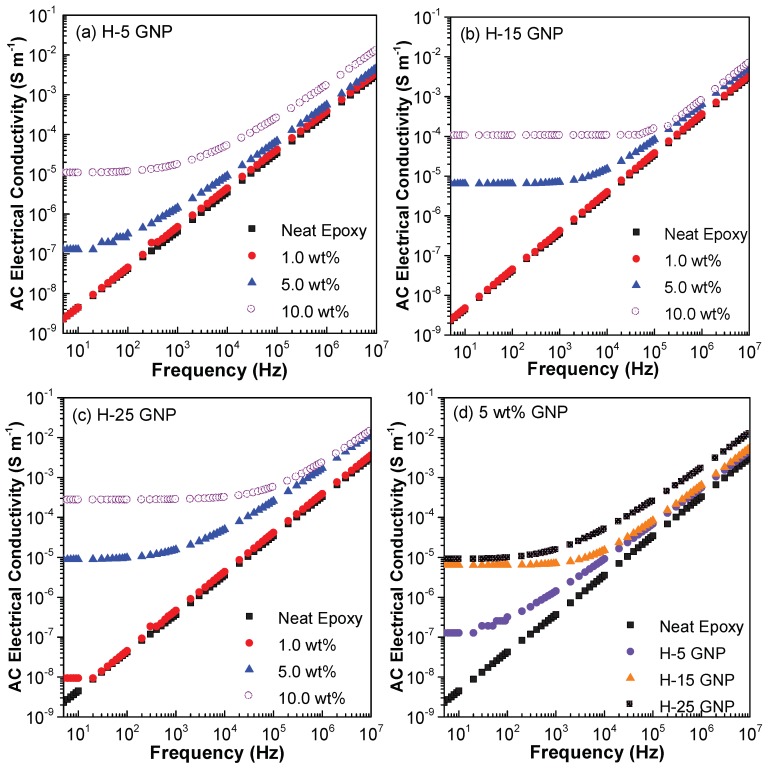
AC electrical conductivity of GNP/epoxy nanocomposites (**a**) GNP H-5; (**b**) GNP H-15; (**c**) GNP H-25; (**d**) *f*_GNP_ = 5 wt %.

Figure 10 plots the variations of AC electrical conductivity with frequency at different GNP concentrations. For all GNP sizes, no significant improvement in the AC electrical conductivity has been found at 1.0 wt % for the given frequency range. However, significant enhancement of AC electrical conductivity is found at lower AC frequency for the nanocomposites with 5 wt % and 10 wt % of GNPs. The comparisons among the four figures advise that the increase of the GNP size is favorable to increase the electrical conductivity. This is because with fixed thickness, increased diameter of GNP corresponds to larger aspect ratio, leading to increased density of percolation networks as reported in epoxy systems [71,72]. In addition, the comparisons in Figure 10d suggests that at lower frequency (i.e., less than 100 Hz), the electrical conductivity increases dramatically when GNP diameter increases from 5 µm to larger size, while limited discrepancy is observed between the results with diameters being 15 µm and 25 µm, respectively. As the AC frequency further increases, the variation of the electrical conductivity becomes less sensitive to GNP size. 

**Figure 11:**
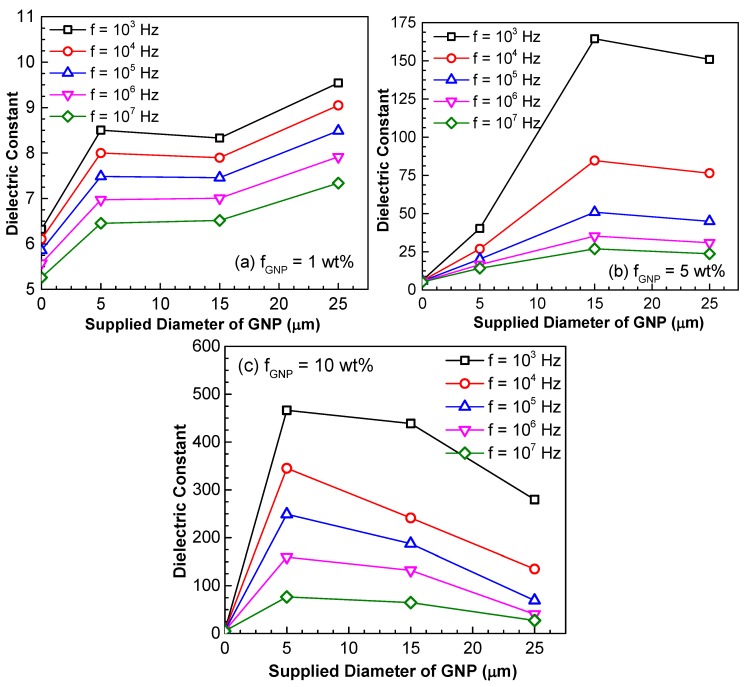
Dielectric constant of GNP/epoxy nanocomposites (**a**) *f*_GNP_ = 1.0 wt %; (**b**) *f*_GNP_ = 5.0 wt %; (**c**) *f*_GNP_ = 10.0 wt %.

**Figure 12:**
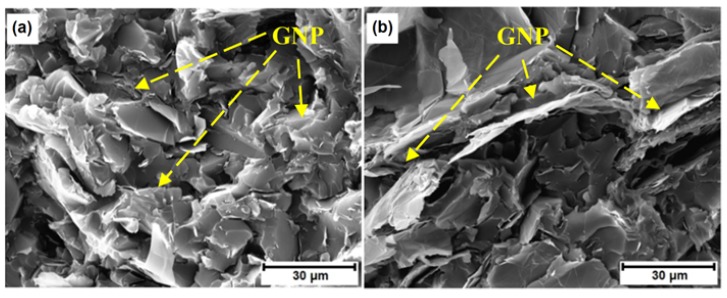

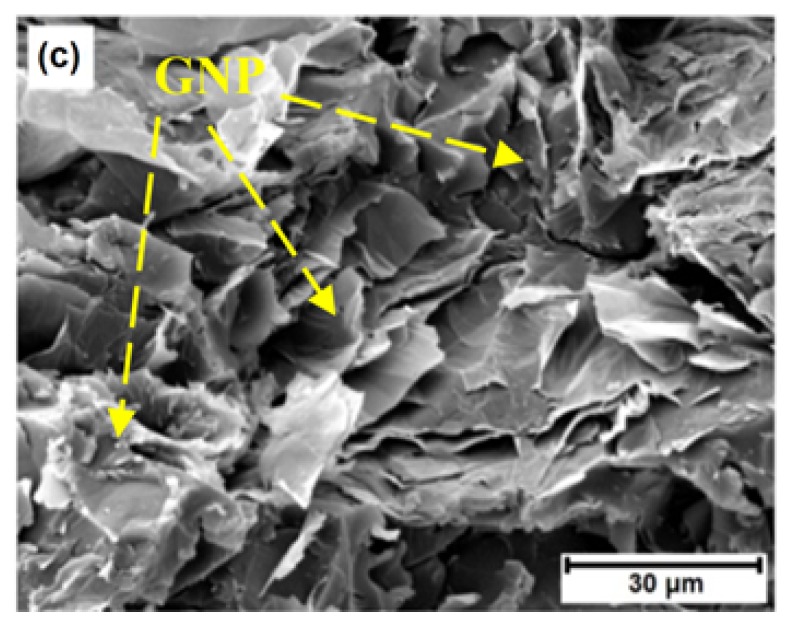
SEM of fracture surfaces of GNP/epoxy nanocomposites with 10.0 wt % (**a**) GNP H-5; (**b**) GNP H-15; (**c**) GNP H-25.

**Figure 13:**
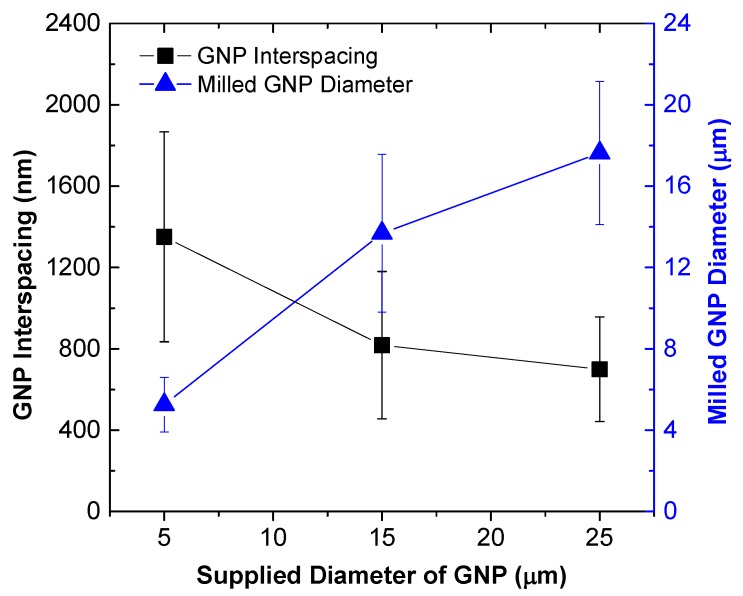
GNP interspacing and diameter after milling at varying supplied GNP diameters (i.e., H-series) in epoxy nanocomposite containing 10 wt % GNP.

The effect of GNP size on the dielectric constant is demonstrated in Figure 11, in which three weight fractions, i.e., 1 wt %, 5 wt % and 10 wt % GNP, are considered. Again, it is observed that the increase of the AC frequency results in the drop of the dielectric constant. When the GNP weight fraction is lower, i.e., 1 wt %, the dielectric constant increases with the increase of the size of the GNPs. However, when the weight fraction of GNP increases to 5 wt %, the dielectric constant is found to decrease as the diameter of the GNP is larger than 15 μm. Such a trend of decrease becomes even prominent when the GNP weight fraction increases to 10 wt %, which is indicated by the earlier start of the decrease in dielectric constant (i.e., diameter of GNP is larger than 5 μm). It is well accepted that larger sized conductive fillers are beneficial to form conductive networks. Therefore, the decrease in dielectric constant in Figure 11b,c indicates the nanocomposites are transitioning from insulator to conductor as the GNP size increases. Figure 12 presents the SEM fractographs of the GNP epoxy nanocomposites at 10 wt % GNP. Figure 13 gives the GNP interspacing and milled diameter of GNPs at 10 wt % in epoxy at varying supplied sizes of the GNP. It can be observed that the milled size of the GNPs is far smaller than the supplied GNP diameter. This was attributed to the shearing and size reduction of the GNPs during the three-roll milling process. As expected, with the increase in the milled GNP size, the interfiller spacing reduces. GNPs with larger lateral size can easily form a conductive network because GNPs with a high greater aspect ratio provide longer percolation paths and reduce the number of inter-platelet junctions where electrical resistance increases sharply.

## 4. Conclusions

Three-roll milling process is employed to disperse different series of GNPs in to epoxy. The measured AC electrical conductivity and dielectric constant suggest that the addition of GNP improves the electrical and dielectric properties of the GNP/epoxy nanocomposites. It is found GNPs with larger size and surface area are always favorable reinforcements to increase the electrical conductivity. However, the effects of GNP size and surface area on the dielectric properties are dependent on the applied AC frequency and GNP concentration. At lower GNP concentration, limited improvement in the electrical conductivity is observed, indicating sparse formation of percolated networks among the dispersed GNPs. At higher GNP concentration but lower AC frequency, significant reinforcing effects of GNP on the electrical properties are observed. For higher AC frequency, all curves for electrical conductivity and dielectric constant tend to merge together with limited discrepancy. This observed phenomenon can be attributed to the dependency of the electron hopping between two neighboring GNPs on AC frequency. Table 1 presents a comparison of the dielectric constant and AC electrical conductivity of the epoxy GNP nanocomposites at an applied frequency of 10^3^ Hz for various (a) GNP contents, (b) supplied GNP surface areas, and (c) supplied GNP diameters. Indeed, it is evident that the magnitude of improvement in both the dielectric constant and AC electrical compared to the unmodified epoxy is highly sensitive with increase in the GNP diameter. 

Findings of the present study may provide design considerations for tailoring the appropriate or desired electrical properties for epoxy used in engineering structures and coatings. Considering the variation in geometric properties of GNPs and correlating with the electrical properties, further work shall be conducted on investigating the quasi-static and dynamic mechanical properties of the GNP epoxy composites, such as the Young’s modulus, fracture toughness, storage modulus and fatigue delamination resistance. 

## Figures and Tables

**Table 1:** Dielectric constant and AC electrical conductivity properties of epoxy GNP composites at an applied frequency of 10^3^ Hz.

Sample	Dielectric Constant	Improvement (%)	AC Electrical Conductivity (S m^−1^)	Improvement (%)
***Effect of GNP (C-300 series) content in epoxy***				
Neat Epoxy (unmodified)	6.31 (±0.12)	-	3.69 × 10^−7^ (±0.29 × 10^−7^)	-
0.10 wt % GNP	6.31 (±0.14)	-	3.69 × 10^−7^ (±0.14 × 10^−7^)	-
0.50 wt % GNP	6.44 (±0.07)	2.0%	4.05 × 10^−7^ (±0.11 × 10^−7^)	9.9%
1.00 wt % GNP	6.69 (±0.13)	5.9%	4.24 × 10^−7^ (±0.18 × 10^−7^)	15.0%
1.50 wt % GNP	6.85 (±0.11)	8.4%	4.31 × 10^−7^ (±0.32 × 10^−7^)	16.9%
2.00 wt % GNP	7.18 (±0.08)	13.6%	4.56 × 10^−7^ (±0.12 × 10^−7^)	23.5%
5.00 wt % GNP	7.32 (±0.08)	16.1%	5.19 × 10^−7^ (±0.25 × 10^−7^)	40.6%
10.0 wt % GNP	20.2 (±0.35)	220%	1.25 × 10^−6^ (±0.08 × 10^−6^)	239.5%
20.0 wt % GNP	81.1 (±0.91)	1180%	6.84 × 10^−6^ (±0.11 × 10^−6^)	1750%
***Effect of supplied GNP surface area (C-series) at 10 wt % in epoxy***				
Neat Epoxy (unmodified)	6.31 (±0.12)	-	3.69 × 10^−7^ (±0.29 × 10^−7^)	-
300 m^2^ g^−1^ GNP	20.2 (±0.41)	220%	1.25 × 10^−6^ (±0.08 × 10^−6^)	240%
500 m^2^ g^−1^ GNP	36.1 (±0.85)	470%	4.29 × 10^−6^ (±0.13 × 10^−6^)	1060%
750 m^2^ g^−1^ GNP	32.7 (±0.61)	420%	8.76 × 10^−6^ (±0.12 × 10^−6^)	2270%
***Effect of supplied GNP diameter (H-series) at 10 wt % in epoxy***				
Neat Epoxy (unmodified)	6.31 (±0.12)	-	3.69 × 10^−7^ (±0.29 × 10^−7^)	-
5 µm	467 (±2.8)	7280%	1.75 × 10^−5^ (±0.14 × 10^−5^)	4630%
15 µm	438 (±3.1)	6840%	1.05 × 10^−4^ (±0.21 × 10^−4^)	28,500%
25 µm	280 (±2.7)	4320%	2.80 × 10^−4^ (±0.17 × 10^−4^)	75,800%
